# Measuring strategic control in implicit learning: how and why?

**DOI:** 10.3389/fpsyg.2015.01455

**Published:** 2015-09-24

**Authors:** Elisabeth Norman

**Affiliations:** Department of Psychosocial Science, Faculty of Psychology, University of BergenBergen, Norway

**Keywords:** implicit learning, artificial grammar learning, serial reaction time task, strategic control, flexibility, control, process dissociation procedure, task switching

## Abstract

Several methods have been developed for measuring the extent to which implicitly learned knowledge can be applied in a strategic, flexible manner. Examples include generation exclusion tasks in Serial Reaction Time (SRT) learning (Goschke, 1998; Destrebecqz and Cleeremans, 2001) and 2-grammar classification tasks in Artificial Grammar Learning (AGL; Dienes et al., 1995; Norman et al., 2011). Strategic control has traditionally been used as a criterion for determining whether acquired knowledge is conscious or unconscious, or which properties of knowledge are consciously available. In this paper I first summarize existing methods that have been developed for measuring strategic control in the SRT and AGL tasks. I then address some methodological and theoretical questions. Methodological questions concern choice of task, whether the measurement reflects inhibitory control or task switching, and whether or not strategic control should be measured on a trial-by-trial basis. Theoretical questions concern the rationale for including measurement of strategic control, what form of knowledge is strategically controlled, and how strategic control can be combined with subjective awareness measures.

## Introduction

Strategic control over knowledge refers to whether it can be deliberately applied or withheld according to instructions (Jacoby, 1991). In contrast, knowledge that influences behavior regardless of the person’s intentions is considered to have an automatic influence. Strategic control is traditionally regarded as a defining property of consciousness (Jacoby, 1991; see also Seth et al., 2008).

Most procedures for measuring strategic control in implicit cognition have been inspired by the Process Dissociation Procedure (PDP), which is a procedure originally developed to estimate the relative influence of strategic versus automatic processes by comparing performance under conditions where the person “tries to” versus “tries not to” engage in some act, referred to as “opposition logic” (Jacoby, 1991). Measures based on the PDP are most often used to assess the relative influence of conscious and unconscious knowledge. The aim of this paper is to address and discuss some methodological and theoretical questions related to the measurement of strategic control in implicit learning, where learning of complex stimulus regularities occurs in the absence of a conscious intention to learn or full conscious awareness of the acquired knowledge. The discussion will focus on the two most well-known implicit learning paradigms, namely the Serial Reaction Time (SRT) task and the Artificial Grammar Learning (AGL) task.

## Examples of Strategic Control Measurement in Implicit Learning

### The Serial Reaction Time Task

In the SRT task, participants are trained to make fast motor responses to a visual target that moves between positions on a computer screen according to a complex, pre-defined sequence. Learning is measured in terms of reaction time differences between target movements that follow versus violate this sequence (Nissen and Bullemer, 1987).

Strategic control would imply that people can intentionally apply sequence knowledge in line with instructions. One adaptation of the PDP to sequence learning is to instruct participants to generate a sequence that does *not* contain the regularities seen during training. Goschke (1998), and later Destrebecqz and Cleeremans (2001), refer to this as the generation *exclusion* task. In what the same authors refer to as the generation *inclusion* task participants are simply instructed to generate a sequence that is as similar to the training sequence as possible. If participants reproduce fewer trained sequence regularities under exclusion than under inclusion instructions, this is taken to indicate strategic control^1^. On each trial of a *cued generation task*, participants are first presented with a short sequence of, e.g., 5 (Wilkinson and Shanks, 2004) or 2 (Fu et al., 2008) elements, and then asked to indicate a continuation response that either follows the sequence regularity (i.e., inclusion instructions) or violates it (i.e., exclusion instructions).

A different form of a cued generation task is the *generation rotation task* (Norman et al., 2007). Stimuli are presented in a square layout. On each trial the participant predicts the next target position, but indicates it by rotating their response, clockwise or anti-clockwise, in accordance with a randomly varying post-trial cue, i.e., the numbers +1, –1, or –2. Performance is compared to a *direct* version of the same task.

Mong et al. (2012) introduced an SRT procedure where all participants learn two different sequences. Participants are then presented with a series of short sequences that they are asked to classify according to familiarity. Inclusion instructions are to classify sequences as “old” if they follow either regularity. Exclusion instructions are to classify a sequence as “old” if it follows their target sequence, and to respond “new” if not.

### The Artificial Grammar Learning Task

In the training phase of an AGL task, participants are presented with a series of non-word letter strings in which the selection and ordering of letters are governed by a complex, finite-state grammar of which they are not informed (Reber, 1967). Learning is measured as the ability to classify novel letter strings according to grammaticality. Here, strategic control would refer to the extent to which the person was able to apply or withhold grammar knowledge. It is typically measured in a two-grammar situation, where participants are initially exposed to two sets of letter strings governed by different grammars (A vs. B; Dienes et al., 1995). On each trial of a subsequent test phase, letter strings following these two grammars are intermixed with ungrammatical strings, and the instruction is to classify letter strings according to their target grammar, i.e., either Grammar A or Grammar B.

Norman et al. (2011) referred to this as a *pure-block* procedure, and developed the alternative *mixed-block procedure.* On each test trial, participants are presented with three letter strings, one ungrammatical, and one from each of two trained grammars (A or B). Whether they are to identify A or B varies randomly between test trials.

Higham et al.’s (2000) procedure also involves two training phases. In the test phase, participants are presented with a list of all test stimuli. Participants who receive so-called *in-concert* instructions are asked to rate any strings consistent with either grammar as “grammatical”, whereas those who receive *opposition* instructions are asked to rate only those items consistent with one of the grammars as grammatical, assumed to require strategic control.

Within statistical learning^2^, Franco et al. (2011) have developed a procedure where participants hear two speech streams generated from two “artificial languages” (L1 and L2). In a discrimination task participants are presented with words from L1, L2, or neither. Under inclusion instructions they are to say “yes” if the word is from either language, and under exclusion instructions they are to say “yes” if it is from their target language (L1 or L2).

## Methodological Questions

### Type of Task

As we see from the above examples, there is large variation in the way strategic control has been measured with the SRT and AGL tasks, both in terms of in which part of the experiment it is measured, the type of judgment it involves, and whether it allows for an independent measure of learning.

In AGL the strategic control measure is incorporated into the classification task itself, i.e., it is measured within the same judgment as learning. In SRT, strategic control is most often measured in a separate generation task after the training phase is completed. Mong et al.’s (2012) recognition task is also conducted in a separate phase.

There is also variation in the type of judgment the strategic control measurement requires. Whereas a recognition task involves making a series of forced-choice judgments to the presented sequences, a generation task requires participants to actively produce sequence elements. Moreover, whereas free generation requires the person to produce a long (e.g., 96 element) string of responses, each trial of a cued generation task requires one response only. This procedural variation could in principle reduce the comparability of strategic control measurement across different experimental paradigms and procedures.

In SRT but not AGL it is possible to estimate learning by RT data, independently of strategic control. This may be relevant if one wants to analyze data only from participants who show learning, or compare strategic control for different degrees of learning. Even in paradigms that do not allow for a completely independent measure of learning, it is still possible to identify subgroups based on learning performance. For instance, Franco et al. (2011) separated between “low” and “high” learners based on inclusion scores. In 2-sequence SRT tasks it may also be of interest to control for possible differences in the amount of learning for the two rules.

### Inhibitory Control or Task Switching?

In SRT tasks there is normally only one sequence to be learned, and strategic control is operationalized as the ability to withhold the influence of this sequence when so instructed. In AGL strategic control is normally studied in 2-grammar designs, a logic which Mong et al. (2012) have applied to SRT and Franco et al. (2011) to statistical learning.

An important question is then whether strategic control over the application of 1 vs. 2 rules involves the same or different mechanisms. This relates to the distinction between *inhibition* and *task switching.* Within working memory research, the two are most often seen as distinguishable executive components (Baddeley, 1996), that may even be inversely related at the level of the individual (Friedman et al., 2011). More specifically, whereas task switching may depend on the ability to clear working memory contents and prior goals when a task changes, inhibition may involve motor response inhibition and/or interference control (Blackwell et al. (2014). Therefore, one cannot rule out the possibility that the ability to flexibly apply two implicitly learned rules may reflect a different form or degree of cognitive control than the ability to inhibit the influence of one implicitly learned rule.

### Global vs. Trial-by-trial Measurement

Some would argue that the ability to deliberately apply or withhold information according to instructions also implies being able to comply with *shifting* instructions. This would be in line with Baars’s (1988) global workspace model, where the ability to apply knowledge in a contextually flexible way is seen as indicative of consciousness. However, traditional exclusion procedures in SRT, and pure-block classification procedures in AGL, both require participants to comply with the same instruction throughout the task. Similarly, in Higham et al.’s (2000) classification procedure the task set is identical across all test items. It could be argued that successful performance in such settings merely requires a global voluntary inhibition of the influence of acquired knowledge rather than a moment-to-moment ability to control the application of this knowledge. An alternative would be to let the instruction change between individual trials, as in the *rotation task* in the SRT task (Norman et al., 2007) or the *mixed-block* procedure in AGL (Norman et al., 2011). Alternating the SRT stimulus-response mapping or the AGL target grammar from test trial to test trial could be seen as a more demanding test of strategic control than instructing participants to activate one mental set and inhibit another at the start of a block of trials. In the task switching literature (Monsell, 2003), the *mixing cost* refers to the difference in performance between blocks of trials where participants apply the same task set across several trials, and mixed blocks, where participants are instructed to alternate between which of *n* rules to apply. Whereas a single task can be executed quite automatically, alternating between multiple tasks, each involving a different instruction, is assumed to demand more executive control.

## Theoretical Questions

### What does Strategic Control Imply?

Traditionally strategic control has been regarded as a criterion for deciding whether or not knowledge is consciously available (e.g., Wilkinson and Shanks, 2004). It can also be used to identify the *conditions* under which learning is associated with more conscious or unconscious knowledge. For instance, Destrebecqz and Cleeremans (2001) reported that participants could exclude under certain response-stimulus intervals but not others, and Franco et al. (2011) found exclusion performance to depend on the individual’s level of learning. Similarly, in Fu et al. (2008) study, providing participants with a reward incentive increased exclusion performance.

Others have used strategic control as one of several measures to assess the properties of different *types of knowledge* assumed to result from implicit learning. For example, strategic control has been combined with other measures in order to identify knowledge states that correspond to *fringe consciousness*, defined as consciously available feelings that occur in relationship to unconscious knowledge (Norman et al., 2006, 2007, 2010, 2011). In a context where the nature of the rule is made less salient, e.g., by introducing random variation in irrelevant stimulus properties from one stimulus to the next, some participants may develop incorrect hypotheses about the nature of the rule. For instance, they may report that they think target positions in SRT were predicted by color combinations rather than previous target positions. If these *unaware* participants nevertheless show strategic control over the sequence (e.g., on a generation rotation task), this could be taken to indicate that they have conscious access to a feeling that reflects unconscious knowledge of the sequence. A similar logic may be applied to AGL (see Norman et al., 2011).

Similarly, Wan et al. (2008) looked at whether strategic control in AGL differed depending on the extent to which *judgment knowledge* of whether a certain string follows the grammatical structure, versus *structural knowledge* of the grammar rules, were conscious of unconscious (see Dienes and Scott, 2005, for an introduction to this distinction and related self-report measures). Significant strategic control was found even for trials attributed to strategies not involving conscious structural knowledge, i.e., familiarity, intuition, and random choice. Similarly, Fu et al. (2010) found successful exclusion performance on generation trials that were attributed to intuition, rules, and memory, but not to random choice. Whereas intuition is assumed to involve conscious judgment knowledge but not conscious structural knowledge, rules and memory attributions are taken to indicate that both forms of knowledge are conscious (Dienes and Scott, 2005). Fu et al. (2010) therefore took this result to indicate that strategic control can be based on both conscious and unconscious structural knowledge. Here, we see that measurement of strategic control, when complemented with subjective awareness measures, can contribute to a more detailed understanding of the subtypes of knowledge acquired in implicit learning and their properties.

### Strategic Control Over What?

A related question is what kind of knowledge or signal people are strategically controling in implicit learning situations.

One possibility is that people are strategically controling grammar/sequence knowledge itself. Following Baars (1988) and Jacoby (1991), strategic control could then be taken to indicate that learning is fully conscious. However, recent studies within other areas of implicit cognition have provided evidence suggesting that unconscious knowledge can be strategically applied (Lau and Passingham, 2007; Schmidt et al., 2007; van Gaal et al., 2010; van Gaal and Lamme, 2012). The question then is whether and in what way unconscious knowledge may be associated with strategic control.

Within the *fringe consciousness* framework (Norman et al., 2006, 2007, 2011) it could be argued that what participants are strategically controling is a feeling that reflects knowledge of the underlying rule, even if this knowledge is not itself consciously represented. In SRT, such feelings could take the form of certain positions “feeling right”, and in AGL of certain letter strings feeling more familiar or coherent than others. If such feelings can be used flexibly in accordance with shifting task demands this could be taken to indicate that the feeling itself, rather than the underlying knowledge of the learned structure, is conscious. In terms of the *judgment* vs. *structural knowledge* distinction (Dienes and Scott, 2005), “unconscious control” could be understood as occuring when people are not conscious of structural properties of the grammar or sequence. In such situations they may still be conscious of whether they think a string is familiar or not, or whether it feels like their generation responses comply with the sequence.

### Strategic Control and Subjective Awareness Measures

Even though strategic control is traditionally regarded as a criterion for deciding whether acquired knowledge is conscious or not, it has been argued that strategic control used as a sole measure of consciousness may lead to a distorted picture of what people are consciously aware of in implicit learning, as pointed out by Gaillard et al. (2014):

“… defining consciousness as the ability to exert intentional control over the acquired knowledge fails, however, to capture one of its essential aspects, that is, the qualitative, subjective or first-order properties of conscious experience.” (ibid., p. 53).

The importance of supplementing measurement of strategic control with subjective awareness measures also follows from the theoretical perspectives presented above, in which consciously experienced, fringe feelings are seen to reflect unconscious rule knowledge, or implicit learning is hypothesized to give rise to different forms of knowledge that may converge or dissociate in terms of consciousness. For example, in order to establish that knowledge corresponds to fringe consciousness, one needs systematic ways of measuring people’s degree of rule awareness (Norman et al., 2007, 2011). To assess whether judgment vs. structural knowledge is conscious or unconscious, self-reported decision strategy judgments can be collected for every classification or generation response (Dienes and Scott, 2005; Scott and Dienes, 2008). Other awareness measures include confidence ratings (Norman and Price, 2015), recognition ratings (Destrebecqz and Cleeremans, 2001), and post-decision wagering (Wierzchon et al., 2012).

An additional way in which subjective awareness measures can be applied is to assess people’s metacognitive awareness of their degree of strategic control. For example, Destrebecqz and Cleeremans (2003) found that participants were more confident in exclusion than inclusion performance, that confidence was related to accuracy only for inclusion, and only in the highest RSI condition. Similarly, Norman et al. (2007) found a tendency for confidence to more accurately reflect accuracy on rotation than direct generation. Thus, this form of measurement may be used to start exploring to what extent participants have metacognitive insight into their flexible application of rules that are in themselves not necessarily conscious (see also Mealor et al., 2014).

## Conclusion

I have outlined how strategic control measurement can provide us with a better understanding of the properties of knowledge acquired through implicit learning, and I have pointed to some methodological and theoretical considerations that researchers should take into account. Importantly, I have argued that strategic control may be applied not only over conscious sequence/grammar knowledge, but also over conscious feelings that relate to acquired knowledge that in itself is not consciously accessible. Therefore measures of strategic control should not be considered as awareness measures *per se* but should be complemented with subjective measures.

A better understanding of how and to what extent implicitly learned knowledge can be strategically controlled may provide an important contribution to our knowledge of implicit learning, our knowledge on consciousness more generally and finally, to our understanding of how implicitly learned knowledge may influence behavior in everyday situations (Norman and Price, 2012).

## Conflict of Interest Statement

The author declares that the research was conducted in the absence of any commercial or financial relationships that could be construed as a potential conflict of interest.

## Footnotes

1 The difference, under exclusion instructions, in the amount of trained regularities versus regularities following an untrained sequence (i.e., *baseline* performance) under exclusion instructions, can be regarded as an estimate of automatic influence.

2 Statistical learning can be seen as a form of implicit learning. Even though it is not a form of SRT or AGL, I have chosen to include it in the paper due to its methodological relevance, and placed it in the AGL section because this particular experiment is concerned learning of artificial languages.
